# *Coxiella burnetii* in ruminants from Somalia

**DOI:** 10.1007/s11250-025-04649-4

**Published:** 2025-09-22

**Authors:** Aamir M. Osman, Ahmed A. Hassan-Kadle, Igor S. Silito, Caroline Tostes Secato, Abdalla M. Ibrahim, Maria Carolina A. Serpa, Marcos R. André, Thállitha S. W. J. Vieira, Marcelo B. Labruna, Rosangela Z. Machado, Rafael F. C. Vieira

**Affiliations:** 1 Graduate Program on Veterinary Sciences, Universidade Federal do Paraná, Curitiba, Paraná Brazil; 2 Somali One Health Centre, Abrar University, Mogadishu, Somalia; 3 Department of Animal Health and Veterinary Services, Ministry of Livestock, Forestry, and Range, Mogadishu, Somalia; 4 Abrar Research and Training Centre, Abrar University, Mogadishu, Somalia; 5 Departamento de Medicina Veterinária Preventiva e Saúde Animal, Faculdade de Medicina Veterinária e Zootecnia, Universidade de São Paulo, São Paulo, SP Brazil; 6 Vector-Borne Bioagents Laboratory (VBBL), Department of Pathology, Reproduction and One Health, Faculty of Agrarian and Veterinary Sciences, São Paulo State University (FCAV/UNESP), Jaboticabal, Brazil; 7 Department of Chemistry, The University of North Carolina at Charlotte, Charlotte, USA; 8 Center for Computational Intelligence to Predict Health and Environmental Risks (CIPHER), The University of North Carolina at Charlotte, Charlotte, USA; 9 Department of Epidemiology and Community Health, The University of North Carolina at Charlotte, Charlotte, USA

**Keywords:** Q fever, Coxiellosis, Livestock, One health, Sub-Saharan Africa

## Abstract

Q fever in humans and coxiellosis in ruminants are caused by *Coxiella burnetii*, a zoonotic obligate intracellular bacterium. This infection poses significant health and economic risk. Effective control strategies rely on a thorough understanding of their epidemiology. In this study, we examined the prevalence of *C. burnetii* in 372 ruminants (190 goats, 133 cattle, and 49 sheep) from the Lower Shabelle and Benadir Regions of Somalia. Diagnostic testing was performed using a quantitative PCR (qPCR) assay that targets the bacterium’s repetitive element *IS111* and the Indirect Fluorescent Antibody Test (IFAT) against the *C. burnetii* strain At12. A total of 47 out of 372 (12.6%) ruminants tested positive for anti-*Coxiella burnetii* IgG antibodies, with seropositivity detected in 40 out of 190 goats (21.1%) with endpoint titers ranging from 64 to 2,048, and 7 out of 49 sheep (14.3%) with endpoint titers ranging from 64 to 8,192, while all cattle tested negative. Notably, only one goat that was seropositive also tested positive for *C. burnetii* by qPCR, while all other ruminants were negative. These findings provide evidence of the presence of *C. burnetii* in Somali livestock, emphasizing its public health risk and highlighting the necessity for targeted surveillance and control strategies to prevent zoonotic transmission.

## Introduction

Q fever, an emerging yet neglected zoonotic disease, poses a significant public health threat and leads to substantial economic losses worldwide. It is caused by *Coxiella burnetii (C. burnetii)*, an aerobic, intracellular, Gram-negative bacterium (Ullah et al. 2022; Sahu et al. 2020). Humans typically acquire infection through airborne transmission or direct contact with infected animals, especially their reproductive tissues or animal-derived products (ECDC 2010). Domestic ruminants serve as the main reservoirs of *C. burnetii*, although other domestic and wild animals including pets, rabbits, and birds may also play a role in its transmission to humans (WOAH 2018). Despite its zoonotic potential, there is no substantial evidence of human-to-animal transmission under natural conditions. Infected animals often experience reproductive complications such as infertility, endometritis, stillbirths, and late-term abortions, contributing to extensive bacterial shedding in the environment (Rodolakis et al. 2007; WOAH 2018; Eldin et al. 2017).

Ticks can transmit *C. burnetii*, however the likelihood of transmission depends on several factors, including the bacterial load in the tick and the extent to which infected ruminants shed the bacterium into the environment through birth products, milk, feces, or urine (Guatteo et al. 2011; Cardinale et al. 2014). Humans become infected primarily through inhalation of contaminated aerosols, contact with infected animals, consumption of unpasteurized dairy products, their bodily fluids, and tick bites (Njeru et al. 2016; Bernard et al. 2012). Given that Q fever is listed in the Terrestrial Animal Health Code of the World Organization for Animal Health (WOAH), outbreaks must be reported by member nations (WOAH 2018). Additionally, the classification of *C. burnetii* as a potential bioterrorism agent by the Centers for Diseases Control and Prevention (CDC) has made the disease reportable in many countries (Bernard et al. 2012).

Q fever is a significant occupational zoonotic disease with considerable public health and socioeconomic implications. It primarily affects individuals who work closely with animals, including researchers, livestock owners, abattoir workers, and veterinarians (Robi et al. 2023). This zoonotic disease presents a wide clinical spectrum in humans, from fever and pneumonia to more severe conditions like hepatitis. Certain groups especially pregnant women and immunocompromised individuals are at increased risk of serious complications, including endocarditis and stillbirths (Angelakis and Raoult 2010). Ticks have long been suspected to play a role in the natural transmission cycle of *C. burnetii*, particularly those belonging to the genera Ixodes, *Rhipicephalus*,* Amblyomma*, and *Dermacentor*. Over 70 tick species have been reported to harbor *Coxiella* organisms (Getachew et al. 2024; Kazar 2005). However, recent evidence suggests that many of these reports on ticks might be related to *Coxiella-*like endosymbionts rather than to the pathogen *C. burnetii* (Duron et al. 2015). Despite this, several studies have confirmed the presence of *C. burnetii* in ticks, supporting their potential role in disease transmission (Parker and Davis 1938; Pacheco et al. 2013; Körner et al. 2021; Buysse et al. 2021).

In Somalia, *Rhipicephalus* and *Amblyomma* are the most prevalent tick genera, with 13 species of *Rhipicephalus* and six species of *Amblyomma* documented (Collere et al. 2024; Ferrari et al. 2022; Frangoulidis et al. 2021).

Previous studies have revealed a concerning picture regarding Q fever in Somalia where a retrospective study reported a high seroprevalence rate of 37% among adult humans (Botros et al. 1995). Recently, 59% of ticks collected from camels in the northeastern part of the country Puntland tested positive for *C. burnetii* and *Coxiella*-like endosymbionts (CLE) by *IS1111*-PCR (Frangoulidis et al. 2021). These findings suggest a significant zoonotic risk, likely driven by reservoirs in livestock populations. Despite this, there has been no assessment of *C. burnetii* in ruminants such as sheep, goats, and cattle which are known to be important reservoirs for the bacterium and may play a critical role in its transmission dynamics in Somalia. Traditional practices in Somalia, such as multi-grazing systems, handling aborted animals with bare hands, and consuming raw milk, significantly elevate the risk of zoonotic transmission. These practices, combined with the potential impact of *C. burnetii* on livestock productivity, pose serious socioeconomic and health challenges, mainly in resource-limited, livestock-dependent communities (Vanderburg et al. 2014). Taken together, these public and animal health concerns make Q fever a priority for public policymakers and the food industry, especially in animal production. Understanding the actual prevalence of *Coxiella* infection is crucial for better risk assessment and will help policymakers and veterinarians design effective control programs which are currently lacking. Therefore, this study aimed to investigate the prevalence of *C. burnetii* in ruminants in Somalia using serological and molecular methods.

## Materials and methods

### Study design

This cross-sectional study, performed from December 2018 to January 2020 in the Benadir and Lower Shabelle regions of Somalia, examined blood samples from 372 animals, comprising 190 goats, 133 cattle, and 49 sheep, initially collected for investigations on *Bartonella* spp. and hemoplasmas (Osman et al. 2024; Ferrari et al. 2022). For serological testing, 5 mL of blood was drawn into serum separator tubes (BD Vacutainer^®^, Franklin Lakes, NJ, USA), centrifuged at 1500 × g for 5 min to separate the serum, and stored at − 20 °C for further analysis.

### Serological analysis

Serum samples were tested for anti-*C. burnetii* IgG antibodies using an Indirect Fluorescent Antibody Test (IFAT). The IFAT utilized crude antigens derived from the *C. burnetii* strain At12, cultivated in Vero cells with low passage according to established protocols (Pacheco et al. 2013). For each assay, 15 µL of *C. burnetii*-infected cells were added of the 12 wells on microscopic slides for each experiment. Phosphate-buffered saline (PBS) at pH 7.2 was used to serially dilute serum samples in two-fold increments. The dilution ratios ranged from 1:64, 1:128, 1:256, 1:512, 1:1024, 1:2048, 1:4096, and 1: 8192. The wells that had been previously prepared with the antigen were then filled with 20 µL of the diluted serum samples, and they were incubated for 30 min at 37 °C. After two 10-minute PBS washes, the slides were incubated with fluorescein isothiocyanate-labeled rabbit anti-goat (dilution 1:1,500), anti-sheep (dilution 1:1,500), or anti-cattle IgG (dilution 1:2,000) (Sigma, St. Louis, MO, USA). The slides were then washed as previously mentioned, with 1.5 mL of Evans blue added. After drying and mounting the slides on coverslips using buffered glycerin, they were seen at 400x magnification using an Olympus fluorescent microscope (Tokyo, Japan). Positive results were noted for antibody titers of 1:64 or above, and each sample was performed in duplicate (Reeves et al. 2006; Pacheco et al. 2013). For seropositive samples, the endpoint titer was defined as the last dilution in which ≥ 50% of bacterial fluorescence was visible. Negative and positive (which is known to be positive) control sera from goats, sheep, and cattle were also tested at a 1:64 dilution on each slide.

### DNA extraction and real-time quantitative (q)PCR

DNA extraction was performed from 200 µL of EDTA-blood samples and dried blood spots (DBS) on filter paper using a commercial kit (IndiMag^®^ Pathogen Kit, Qiagen for Indical Bioscience, Leipzig, Germany), following the manufacturer’s instructions. To verify successful DNA extraction, a standard PCR targeting the mammalian endogenous gene *glyceraldehyde-3-phosphate dehydrogenase* (*gapdh*) was conducted for each sample (Birkenheuer et al. 2003). Detection of *C. burnetii* DNA was performed using a quantitative real-time PCR (qPCR) assay targeting the *IS1111* element (~ 295 bp) (Klee et al. 2006). The qPCR reaction mixture (final volume: 10 µL) contained 0.2 µM of each primer and hydrolysis probe, 1 µL of DNA (~ 50 ng), PCR buffer (IQ Multiplex Power Mix, BioRad^®^, Hercules, CA, USA), and sterile ultrapure water (Nuclease-Free Water, Promega^®^, Madison, WI, USA). The assay was performed on a Bio-Rad^®^ CFX96 heat cycler using a low-profile multiplate unskirted PCR setup. Standard curves were generated by serially diluting a plasmid containing a 259 bp *C. burnetii IS1111* fragment (pIDTSMART; Integrated DNA Technologies, Coralville, IA, USA) in concentrations ranging from 2.0 × 10⁷ to 2.0 × 10⁰ copies. Each DNA sample was tested in duplicate, and those with a Cq difference greater than 0.5 underwent additional testing.

### Statistical analysis

To analyze the data, SPSS Statistics software^®^ (IBM^®^ Corp, Armonk, NY, USA, version 26) was utilized. The chi-square test was used to determine whether there were significant differences in infection rates between animal species. Results were considered significant when *P* < 0.05. For each variable, the odds ratio (OR), 95% CI, and P-values were computed independently. Epi Info™ software, version 7.2.3.1 (Centers for Disease Control and Prevention, CDC, USA), was used to assemble and analyze the data.

## Results

A total of 47/372 (12.6%, 95% CI: 9.4–16.4) (endpoint titer: 64–8,192) sera reacted to *C. burnetii* antigen. Among these, 40/190 (21.1%, 95% CI: 15.5–27.5) goat serum samples showed reactions to *C. burnetii* antigen (endpoint titer: 64–2,048), followed by 7/49 (14.3%, 95% CI: 5.9–27.2) sheep with endpoint titers ranging from 64 to 8,192. All cattle tested seronegative. Table 1 indicated antibody endpoint titers of the IFAT for *C. burnetii* in ruminants in Somalia. Overall, the median of antibody titers was the same (256) for sheep and goats.

The *gapdh* gene was successfully amplified in all DNA samples. One out of 372 (0.3%) ruminant samples tested positive for *Coxiella* by qPCR. The qPCR performance showed an average efficiency of 98.1% (SD ± 6.5), with an R² of 0.87 (SD ± 0.12), a slope of − 3.378 (SD ± 0.162), and a y-intercept of 37.144 (SD ± 3.5). Among the positive goats, G101 (Cq = 36,5) had an IFA titter of 512. All other animals were qPCR negative for *Coxiella.*

**Table 1:** Antibody endpoint titers of indirect fluorescent antibody test (IFAT) for *Coxiella burnetii* in ruminants in Somalia

Endpoint titers	Cattle [*n* = 0]	Sheep [*n* = 7]	Goat [*n* = 40]
64	0	1 (14.3)	8 (20)
128	0	2 (28.6)	7 (17.5)
256	0	1 (14.3)	9 (22.5)
512	0	1 (14.3)	7 (17.5)
1024	0	0	8 (20)
2048	0	1 (14.3)	1 (2.5)
4096	0	0	0
8192	0	1 (14.3)	0
Median	0	256	256

## Discussion

*Coxiella burnetii*, the causative agent of Q fever, is a zoonotic pathogen that affects both animals and humans (Ullah et al. 2022). In Somalia, the prevalence of *C. burnetii* among ruminants has remained largely unknown, and no control strategies have been implemented to date. Determining the infection status in livestock is essential to elucidate the epidemiological patterns of this bacterium. This study provides the first report on the prevalence of *C. burnetii* in ruminants in Somalia. Serological testing revealed that 12.6% of animals were seropositive, while molecular analysis detected *C. burnetii* DNA in 0.3% of blood samples. These findings are in line with findings from other countries. For instance, studies in Egypt reported seroprevalence rates of 17.4% (Nahed and Khaled 2012) and 18.4% (Selim et al. 2018) using ELISA targeting phase I and II antigens. Similarly, a study in Algeria documented a prevalence of 14.1% using iELISA (Khaled et al. 2016). In contrast, higher rates were observed in Ethiopia (31.6%) and Ghana (21.4%) through ELISA and iELISA, respectively (Getachew et al. 2024; Johnson et al. 2019). The variation in prevalence rates across countries may reflect differences in livestock management practices, climate conditions, tick distribution, sample selection strategies, and the extent of awareness and surveillance systems in place. Furthermore, differences in diagnostic sensitivity between assays should be considered. Across multiple studies, IFAT has shown greater sensitivity compared to ELISA. For example, Wegdam-Blans et al. (2012) reported that IFAT detected IgM phase II antibodies in a significantly higher proportion of acute-phase sera from PCR-confirmed Q fever cases (31.8%) than ELISA (19.7%), and IFAT also demonstrated longer persistence of IgM antibodies up to 12 months post-infection.

Herein, the highest seroprevalence of *C. burnetii* was observed in goats (21.1%), followed by sheep (14.3%). This pattern aligns with findings from Reunion Island, where goats had the highest prevalence (13.4%), followed by cattle (11.8%) and sheep (1.4%) as detected by commercial ELISA phase I and II antigens (Cardinale et al. 2014). However, in contrast to our results, higher seroprevalence were reported in Ghana, Ethiopia and Kenya, where sheep, cattle, and goats showed significantly higher infection rates. For instance, in Ghana, IgG antibodies to *C. burnetii* was detected in 28.4% of sheep, 22% of cattle, and 10% of goats (Johnson et al. 2019), while in Ethiopia, cattle had a seroprevalence of 37.6%, goats 36.7%, and sheep 26.7% (Getachew et al. 2024), and Kenya, 28.3% in cattle, 32% in goats, and 18.2% in sheep (Knobel et al. 2013). In this study, all sampled cattle were seronegative to *C. burnetii*, a finding that contrasts with results from other regions. For instance, studies in Ghana and Ethiopia reported much higher seroprevalence rates in cattle, with 22% and 37.6%, respectively (Johnson et al. 2019; Getachew et al. 2024). Even more remarkable are the findings from Denmark, where cattle had a seroprevalence of 59% (Agger et al. 2010), and South Korea, with a seroprevalence of 10.5% (Seo et al. 2017). Conversely, a large-scale study in Norway found no serological evidence of infection in cattle, highlighting geographical variability (Kampen et al. 2012). The apparent lack of exposure to *C. burnetii* in cattle in our study may be attributed to several local factors, such as differences in environmental conditions, vector presence, and livestock management practices such as limited confinement, lower animal density, or restricted contact with infected birth materials, which may reduce exposure risk in the Somali context. Breed-specific susceptibility could also play a role, as indigenous cattle may differ immunologically from those in other settings. Additionally, testing limitations, including the timing of sample collection relative to infection might also have influenced detection results found herein.

In Somalia, 31 tick species from six genera have been found on domestic animals. With thirteen species, the genus *Rhipicephalus* is the most common, followed by *Hyalomma* with nine and *Amblyomma* with six. In the Greater Horn of Africa, especially in Somalia, *Rhipicephalus pulchellus* is the most common tick species (Pegram 1976; Scaramella 1983; Schoepf et al. 1984; Hassan et al. 2022; Ferrari et al. 2022; Collere et al. 2024). According to Tissot-Dupont et al. (2004), although ticks are known to transmit *C. burnetii*, they are not thought to be the main source of infection for people and domestic animals because the bacteria is typically spread by inhaling dust or aerosols contaminated with the bacteria that sick animals shed. However, ticks can still spread *C. burnetii*, especially to animals that spending extended periods in tick-infested pastures. This is consistent with study from Somalia, where 59.1% of ticks tested positive for *C. burnetii* or *Coxiella*-like endosymbionts in camel-derived ticks (Frangoulidis et al. 2021). Of these, 98.6% were from *Hyalomma* ticks and 1.4% from *Amblyomma* ticks, detected using *IS1111*-based PCR. However, it is critical to note that the *IS1111*-based qPCR assay is one of the least specific markers for detecting *C. burnetii* in CLE-positive ticks (Duron 2015). The use of a target gene with low specificity may lead to an overestimation of the role of ticks as hosts and vectors of *C. burnetii* and overdiagnosis of Q fever in vertebrates (Duron et al. 2015). Furthermore, there is currently no specific method available to reliably distinguish *C. burnetii* from CLE, given the high heterogeneity within the CLE group. Reliable differentiation between *C. burnetii* and CLE requires sequencing conserved genes such as *rrs* or *groEL* (Körner et al. 2021). Although the literature identifies *Rhipicephalus* ticks as the primary carriers of *Coxiella* endosymbionts (Guizzo et al. 2017), the role of *Hyalomma* ticks in this context requires further investigation. The significantly higher prevalence of *Coxiella* in ticks collected from camels compared to our study may be attributed to several factors. First, camels, as semi-nomadic animals, are exposed to diverse ecological zones, particularly in arid and semi-arid regions where tick infestations are prevalent. Camels’ extensive time spent in tick-rich environments increases their likelihood of encountering different ticks. In contrast, goats and sheep may have more limited exposure due to their different grazing patterns and management practices. This is supported by findings from Somalia, where the average number of ticks per goat and sheep was 1.5, compared to 4.4 ticks per camel (Hassan et al. 2022; Collere et al. 2024), respectively. Additionally, camels’ ability to travel long distances across diverse environments expands their interaction with tick populations, further increasing the odds of *C. burnetii* transmission. Moreover, camels’ thick skin and longer lifespans provide an ideal environment for prolonged tick attachment, enhancing the likelihood of bacterial transmission.

In this study, only one out of 372 (0.3%) ruminant samples tested positive for *Coxiella* DNA by qPCR, despite serological evidence of exposure. Blood is not considered an ideal sample type for detecting *C. burnetii* DNA, as the bacterium is typically shed through other biological routes. Future studies should prioritize other biological samples, such as vaginal swabs, abortion products, milk, urine, or feces, to improve detection rates. Studies using these sample types have reported significantly higher prevalence rates. For instance, Trujillo et al. (2023) found that 86.6% of ewes (162/187) were shedding *C. burnetii* DNA through at least one route (vaginal, fecal, or milk). Similarly, Jodełko et al. (2021) used milk samples, vaginal swabs, tissue sections, feces, and placenta as the target biological samples and found that *C. burnetii* DNA was present in 22.2% of sheep flocks and 51.16% of goat herds. The significance of choosing suitable biological samples to precisely determine the prevalence of *C. burnetii* is highlighted by these findings.

Although this study provides important valuable insights into the occurrence of *C. burnetii* in Somali livestock; several limitations should be acknowledged. The limited geographical coverage and small sample sizes for certain animal species introduce sampling bias and may affect the generalizability of the findings. The cross-sectional design further restricts the ability to assess temporal dynamics or establish causal relationships. Additionally, methodological limitation is the exclusive use of blood samples for molecular detection of *C. burnetii*. Blood is not considered the most appropriate sample type for identifying active shedding of the bacterium, which is more commonly excreted through vaginal secretions, milk, feces, and products of abortion. Addressing these limitations in future research is essential for a comprehensive understanding and effective surveillance of Q fever in Somalia within the One Health framework.

In conclusion, this study provides the first report of *C. burnetii* exposure in ruminants in Somalia, highlighting the presence of the pathogen across different species, particularly in goats and sheep. These findings highlight the necessity of focused surveillance and control efforts to reduce the risk of zoonotic transmission and provide important new information about the epidemiology of *C. burnetii* in Somalia. The detection of *C. burnetii* in livestock also raises important public health concerns, particularly for individuals with occupational exposure such as farmers, veterinarians, and abattoir workers. Understanding the role of different livestock species in the transmission of *C. burnetii* will be critical for developing effective public health interventions in the region.

## Data Availability

The data supporting the conclusions of this study can be obtained from the corresponding authors upon reasonable request.
